# Effects of stacking periodicity on the electronic and optical properties of GaAs/AlAs superlattice: a first-principles study

**DOI:** 10.1038/s41598-020-61509-x

**Published:** 2020-03-17

**Authors:** M. Jiang, H. Y. Xiao, S. M. Peng, L. Qiao, G. X. Yang, Z. J. Liu, X. T. Zu

**Affiliations:** 1 School of Physics, University of Electronic Science and Technology of China, Chengdu, 610054 China; 2 Institute of Nuclear Physics and Chemistry, Chinese Academy of Engineering Physics, Mianyang, 621900 China; 3 Department of Physics, Lanzhou City University, Lanzhou, 730070 China

**Keywords:** Structural properties, Electronic structure

## Abstract

The effects of stacking periodicity on the electronic and optical properties of GaAs/AlAs superlattice have been explored by density functional theory calculations. Among the (GaAs)_m_/(AlAs)_m,_ (GaAs)_1_/(AlAs)_m_ and (GaAs)_m_/(AlAs)_1_ (m = 1 to 5) superlattices, the band gaps of (GaAs)_m_/(AlAs)_1_ superlattices decrease significantly as the layer of GaAs increases, and the cut-off wavelengths are found to locate in the near infrared region. For (GaAs)_m_/(AlAs)_1_ SLs, the conduction bands shift toward Fermi level, resulting in the smaller band gap, while conduction bands of (GaAs)_1_/(AlAs)_n_ SLs slightly shift to higher energy, which lead to comparable band gaps. The layer number of GaAs shows negligible effects on the reflectivity spectra of superlattice structures, while the absorption coefficient shows a red-shift with the increasing layer of GaAs, which is beneficial for the application of GaAs/AlAs superlattice in the field of near infrared detector. These results demonstrate that controlling the number of GaAs layers is a good method to engineer the optoelectronic properties of GaAs/AlAs superlattice.

## Introduction

Recently, the novel artificial materials can be engineered on the molecular scale due to the development in the growth techniques (e.g. molecular beam epitaxy and metal-organic vapour phase epitaxy)^1^. The semiconductor (GaAs)_m_/(AlAs)_n_ superlattices (SLs), in which m and n denote the number of stacking periodicity, have been widely applied in various optoelectronic devices, due to their unusual physical properties related to luminescence and optical absorption, etc^2–7^. Despite extensive studies on the electronic and optical properties of GaAs/AlAs SLs, such as band gap and absorption coefficient, there still lacks a comprehensive understanding of the effect of stacking periodicity on the optoelectronic properties of GaAs/AlAs SLs for its application as near infrared detector.

For the (GaAs)_1_/(AlAs)_1_ SL, which consists of one monolayer of GaAs and one monolayer of AlAs, its band gap at room temperature (RT) was determined to be 2.07 eV by an ellipsometry measurement^8^. The peak in resonant Raman scattering at RT has been reported to be at 2.006 eV by Kobayashi *et al*.^9^ and at 2.108 eV by Cardona *et al*.^10^. In the study of low-temperature photoluminescence of (GaAs)_1_/(AlAs)_1_ SL, Jiang *et al*. determined the band gap to be direct with the value of 2.214 eV^11^. Isu *et al*. have used the photoluminescence measurement to investigate the ultrathin-monolayer (GaAs)_m_/(AlAs)_m_ (m = 1~4) SLs and found that the luminescence peak locates at 2.033 eV for (GaAs)_3_/(AlAs)_3_ SL^12^. Theoretically, Zhang *et al*. have studied the band structure for ultrathin (GaAs)_1_/(AlAs)_1_ SL employing first-principles method with a self-energy approach, and its band gap was direct with the value of 2.11 eV^13^. Ferhat *et al*. calculated the energy gap of (GaAs)_1_/(AlAs)_1_ SL employing the empirical pseudopotential method, which was reported to be 2.066 eV^1^. Barkissy *et al*. studied the electronic structures of GaAs/AlAs SL at 4.2 K based on the envelope function formalism^4^. The direct band gap was found to be dependent on the temperature and decreased with the temperature increasing^4^. Botti *et al*. combined density functional theory (DFT) and semi-empirical method to study the band structures of (GaAs)_m_/(AlAs)_m_ SL, who found that for all sizes (m ≥ 1) the band gap was direct^3^. Kahaly *et al*. used full-potential DFT method to investigate the epitaxial GaAs/AlAs SLs, and found the insulating characteristic of the interface^14^.

As for the optical properties of GaAs/AlAs SLs, Hidetoshi *et al*. investigated the optical absorption in a wide temperature range from 77 to 500 K, and a double-peak spectrum at 500 K was found^15^. Moore *et al*. investigated the photoluminescence of GaAs/AlAs SLs at low temperature and its band gap was found to be “pseudo-direct”^16^. Xia employed the tight-binding model to study the dielectric functions of GaAs/AlAs SLs, and found that the dielectronic functions were different from those of bulk crystals, while they were similar to the average results of (GaAs)_m_/(AlAs)_m_ (m ≥ 6) SLs^17^. Tsu *et al*. measured the reflectivity of the GaAs/AlAs SL and reported that the absorption edge for the superlattice was approximately at 1.63 eV^18^. Lou *et al*. have used the macroscopic infrared dielectric tensor to analyze the reflectivity spectra of the GaAs/A1As SL, and their results suggested that the superlattices resembled an anisotropic uniaxial crystal^19^.

**Figure 1:**
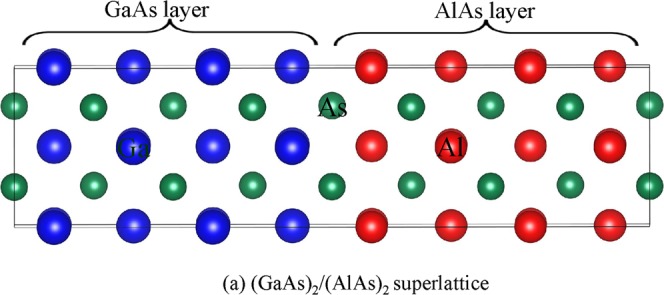
The geometry of (**a**) (GaAs)_2_/(AlAs)_2_ SL. The red, green and blue spheres are the Al, As and Ga atoms, respectively.

In the literature, the researchers generally employed a standard DFT method, which underestimates the band gap of semiconductor SLs. In the present study, we employ a DFT method with reverse scissor correction to explore the optoelectronic properties of GaAs/AlAs SLs (see Fig. 1) in the field of near infrared detector. The results provide deep insights into the variation trend of optoelectronic properties with the stacking periodicity and may have important implications in tuning the electronic and optical properties of GaAs/AlAs SL.

## Results and Discussion

### The ground-state properties of bulk GaAs and AlAs

As shown in Table 1, the lattice constants of bulk AlAs and GaAs are calculated to be 5.64 and 5.63 Å, respectively, which agree well with the available results^3,20^. As for GaAs/AlAs SL, the lattice constant is 5.635 Å, which may result from the negligible lattice mismatch between bulk states. The direct band gap of bulk GaAs is 0.5 eV and the indirect gap of bulk AlAs is 1.31 eV, which are comparable with other calculations^14^. The band gap of AlAs is found to be larger than that of GaAs, which is consistent with experimental results^21^.

**Table 1:** The lattice constant (Å) and band gap (eV) of bulk GaAs and AlAs.

		Lattice constant (Å)	Band gap (eV)
GaAs	Our Cal.	5.63	0.50
	Other Cal.	5.60^a^, 5.61^b^	0.41^c^
	Exp.	5.66^b^	1.41^d^
AlAs	Our Cal.	5.64	1.31
	Other Cal.	5.62^a^, 5.63^b^	1.35^c^
	Exp.	5.62^b^	2.23^d^

^a^Ref. ^3^.

^b^Ref. ^20^.

^c^Ref. ^14^.

^d^Ref. ^21^.

### The electronic properties of GaAs/AlAs superlattices

Based on the optimized structures, the band structures of (GaAs)_m_/(AlAs)_n_ (m, n = 1 to 5) SLs are explored. Considering that the standard DFT method generally underestimates the band gap of materials^22,23^, we calculated the band structure of bulk GaAs, bulk AlAs, and some representative superlattice structures employing DFT with scissor correction^24–27^ and hybrid DFT in the framework of Heyd-Scuseria-Emzefhof (HSE)^28^. The approach of reverse scissor correction is an empirical correction consisting of a shift of the conduction regions up, which has been used to correct the band gap underestimated by DFT, especially to determine the band-gap offsets for interfaces between different semiconductors^22,23,27,29^. The band gap for bulk GaAs, AlAs and (GaAs)_m_/(AlAs)_m_ (m = 1 to 3) SLs obtained from DFT, DFT with scissor correction and hybrid DFT calculations are compared in Table 2. A scissor operator of 0.9 eV is employed, which corrects the band gap of GaAs from 0.5 to 1.40 eV and the band gap of AlAs from 1.31 to 2.21 eV, agreeing well with the experimental values of 1.41 eV and 2.23 eV^21^, respectively. The scissor operator of 0.9 eV is also applied to the (GaAs)_m_/(AlAs)_m_ (m = 1~3) SLs. For (GaAs)_1_/(AlAs)_1_ SL, the corrected band gap of 2.04 eV is in excellent agreement with the experimental value of 2.07 eV^8^. Comparing the band gap obtained from DFT with scissor correction^22,23,27,29^ and hybrid DFT methods^28^, we find that the results compare very well with each other. The DFT with scissor correction method, thus, is employed in the subsequent calculations.

**Table 2:** A comparison of the band gap (eV) for bulk GaAs, AlAs and (GaAs)_m_/(AlAs)_m_ (m = 1 to 3) SLs obtained from DFT, DFT with scissor correction and hybrid DFT calculations.

	Band gap (eV)			
	DFT	DFT with scissor correction	Hybrid DFT	Exp.
GaAs	0.50	1.40	1.44	1.41^a^
AlAs	1.31	2.21	2.16	2.23^a^
(GaAs)_1_/(AlAs)_1_	1.14	2.04	2.06	2.07^b^
(GaAs)_2_/(AlAs)_2_	1.08	1.98	1.99	—
(GaAs)_3_/(AlAs)_3_	1.00	1.90	1.93	—

^a^Ref. ^21^.

^b^Ref. ^8^.

The band gaps obtained from DFT with scissor correction for (GaAs)_m_/(AlAs)_m_ (m = 1 to 5) SLs are presented in Table 3, as compared with the available theoretical results^3,11^. The reverse scissor corrected band gaps of (GaAs)_m_/(AlAs)_m_ SLs (m = 1 to 5) are shown to be direct. Botti *et al*. studied the electronic structures of GaAs/AlAs SLs employing DFT and semi-empirical method, and found that for all sizes (m ≥ 1) the band gaps are direct at the Γ point^3^, which are consistent with our results. It is noted that the value of 1.98 eV for (GaAs)_2_/(AlAs)_2_ SL is slightly larger than the value of 1.76 eV for (GaAs)_5_/(AlAs)_5_ SL, indicating that the band gaps for (GaAs)_m_/(AlAs)_m_ (m = 1 to 5) SLs decrease slowly as the stacking periodicity increases. Correspondingly, the cut-off wavelength of (GaAs)_m_/(AlAs)_m_ (m = 1 to 5) SLs, *λ* = 1240/*E*, increases from 607 to 704 nm, which is located in the near infrared region.

**Table 3:** The band gap (eV) for (GaAs)_m_/(AlAs)_n_ (m, n = 1 to 5) SLs obtained from DFT with scissor correction.

	(GaAs)_m_					
	_m_	1	2	3	4	5
	_n_					
(AlAs)_n_	1	2.04 (1.16^a^, 2.07^b^)	1.81	1.71	1.64	1.6
	2	2.15	1.98 (1.09^a^)	1.84	1.75	1.69
	3	2.14	2.02	1.9 (1.02^a^)	1.81	1.74
	4	2.13	2.01	1.94	1.84	1.76
	5	2.13	2	1.93	1.84	1.76

^a^Ref. ^3^.

^b^Ref. ^8^.

**Figure 2:**
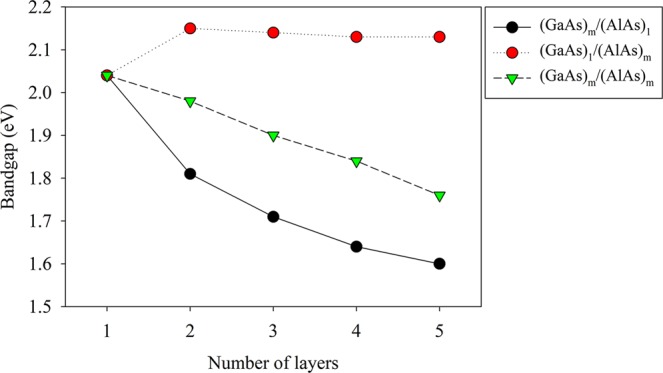
The band gap for (GaAs)_m_/(AlAs)_1_, (GaAs)_1_/(AlAs)_m_ and (GaAs)_m_/(AlAs)_m_ (m = 1 to 5) SLs obtained from DFT with scissor correction as a function of number of layers.

We further analyze the band structures of (GaAs)_m_/(AlAs)_n_ (m ≠ n and m, n = 1 to 5) SLs to investigate how the stacking periodicity affects their electronic properties (see Fig. 2). For the given layers of GaAs, the band gaps of SL structures are comparable with each other, and the cut-off wavelength of (GaAs)_1_/(AlAs)_n_ SLs are all located near 607 nm. Besides, for the given layers of AlAs, the direct band gaps of SL structures decrease monotonously with the increasing layer number of GaAs. It is noted that the direct energy gap of 1.6 eV for (GaAs)_5_/(AlAs)_1_ SL is obviously smaller than the value of 2.04 eV for (GaAs)_1_/(AlAs)_1_ SL. The cut-off wavelength of (GaAs)_m_/(AlAs)_1_ (m = 1 to 5) SLs ranges from 607 to 775 nm. Furthermore, the direct band gap of (GaAs)_10_/(AlAs)_1_, (GaAs)_15_/(AlAs)_1_ and (GaAs)_20_/(AlAs)_1_ SLs obtained from the DFT with reverse scissor correction are determined to be 1.50, 1.46 and 1.44 eV, respectively, and the corresponding cut-off wavelength are calculated to be 826, 849 and 861 nm. These results indicate that controlling the layer number of GaAs is an effective way to tune the cut-off wavelength of GaAs/AlAs SLs in the field of near infrared detectors.

**Figure 3:**
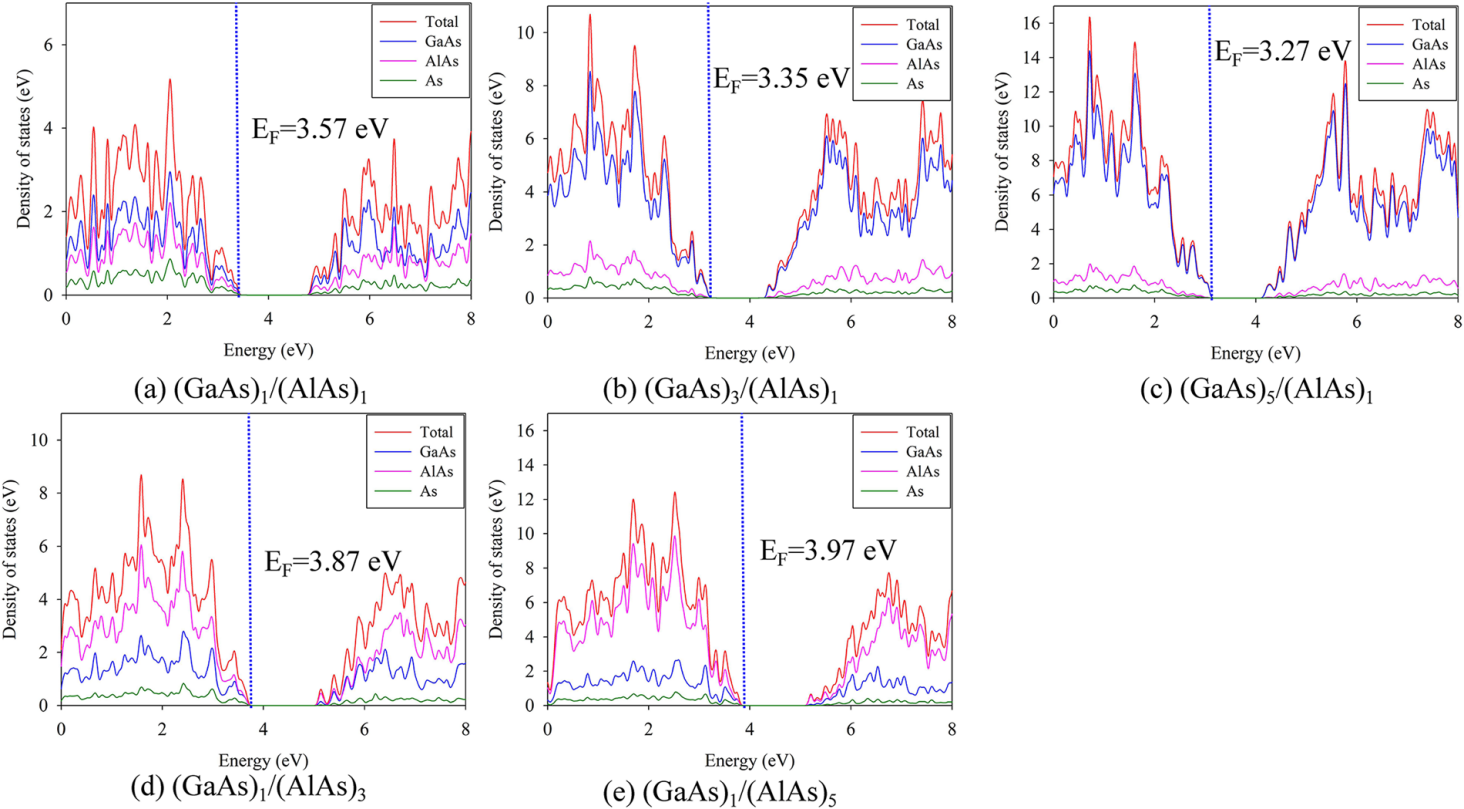
Atomic projected density of state distribution (DOS) for (**a**) (GaAs)_1_/(AlAs)_1_, (**b**) (GaAs)_3_/(AlAs)_1_, (**c**) (GaAs)_5_/(AlAs)_1_, (**d**) (GaAs)_1_/(AlAs)_3_ and (**e**) (GaAs)_1_/(AlAs)_5_ SLs. The Fermi level is indicated by the dashed line.

In order to explore the origin of the influences of the GaAs layer number on the electronic structure of GaAs/AlAs SLs, the density of state distribution (DOS) of (GaAs)_1_/(AlAs)_1_, (GaAs)_3_/(AlAs)_1_, (GaAs)_5_/(AlAs)_1_, (GaAs)_1_/(AlAs)_3_ and (GaAs)_1_/(AlAs)_5_ SLs are analyzed and presented in Fig. 3. As compared with the DOS distribution of (GaAs)_1_/(AlAs)_1_ SL, the Fermi levels for (GaAs)_1_/(AlAs)_n_ and (GaAs)_m_/(AlAs)_1_ SLs shift to higher binding energy level and lower binding energy level, respectively. As for (GaAs)_m_/(AlAs)_1_ SLs, the valence bands are mainly contributed by GaAs (see Fig. 3(a~c)); in the case of (GaAs)_1_/(AlAs)_n_ SLs, the AlAs dominates the valence bands (see Fig. 3(a,d,e)). Also, the conduction bands of (GaAs)_1_/(AlAs)_n_ and (GaAs)_m_/(AlAs)_1_ SLs exhibit different character. For the (GaAs)_m_/(AlAs)_1_ SLs, the conduction bands shift toward the Fermi level, resulting in the smaller band gap, while the conduction bands of (GaAs)_1_/(AlAs)_n_ SLs shift slightly to higher energy, which lead to comparable band gaps. The different variation trend of the band gap of GaAs/AlAs SLs with the increasing layer of GaAs and AlAs, thus, is suggested to originate from their different electronic structures.

### The optical properties of GaAs/AlAs superlattice

As stated above, controlling the layer of GaAs is a good method to tune the band gap and cut-off wavelength of GaAs/AlAs SLs. The effect of GaAs layer number on the absorption coefficient, reflectivity and electron energy loss spectrum of (GaAs)_m_/(AlAs)_1_ (m = 1 to 6) SLs are further investigated. The dielectric function is first calculated

1 $${\rm{\varepsilon }}({\rm{\omega }})={\varepsilon }_{1}({\rm{\omega }})+{\rm{i}}{\varepsilon }_{2}(\omega )$$

where ${\varepsilon }_{1}({\rm{\omega }})$ and ${\varepsilon }_{2}(\omega )$ represent the real and imaginary parts, respectively, and ${\rm{\omega }}$ is phonon energy^30^. The ${\varepsilon }_{2}(\omega )$ is obtained from the momentum matrix elements between the valance and conduction wave functions^30^, i.e.,

2 $${\varepsilon }_{2}(\omega )=\frac{V{e}^{2}}{2\pi \hslash {m}^{2}{\omega }^{2}}\times \int {d}^{3}k{\sum }^{}| < {\varphi }_{c}|p{|{\varphi }_{v} > |}^{2}\delta ({E}_{c}-{E}_{V}-\hslash \omega )$$

where ${\varphi }_{c}$ and ${\varphi }_{v}$ are the wave functions of the conduction and valence bands, respectively, $p$ is the momentum operator, $\hslash$ is the reduced Planck’s constant, $e$ is the electron charge, $V$ is the unit cell volume. The ${\varepsilon }_{1}({\rm{\omega }})$ is calculated by the Kramers-Kroenig relationship^30^:

3 $${\varepsilon }_{1}({\rm{\omega }})=1+\frac{2}{\pi }M{\int }_{0}^{\infty }\frac{{\varepsilon }_{2}({\rm{\omega }}{\prime} ){\rm{\omega }}{\prime} }{{{\rm{\omega }}{\prime} }^{2}-{{\rm{\omega }}}^{2}}d\omega $$

where $M$ represents the principal value of the integral. The absorption coefficient $\,\alpha ({\rm{\omega }})$, reflectivity $R({\rm{\omega }})$ and electron energy loss function $\,L({\rm{\omega }})$ are obtained from the ${\varepsilon }_{1}({\rm{\omega }})$ and ${\varepsilon }_{1}({\rm{\omega }})$ parts:

4 $$\alpha ({\rm{\omega }})=\sqrt{2}\omega {\left[\frac{\sqrt{{\varepsilon }_{1}^{2}-{\varepsilon }_{2}^{2}-{\varepsilon }_{1}}}{2}\right]}^{\frac{1}{2}}$$

5 $$R({\rm{\omega }})=\frac{{(n-1)}^{2}+{k}^{2}}{{(n+1)}^{2}+{k}^{2}}$$

6 $${\rm{L}}({\rm{\omega }})=Im(\frac{-1}{\varepsilon (\omega )}).$$

**Figure 4:**
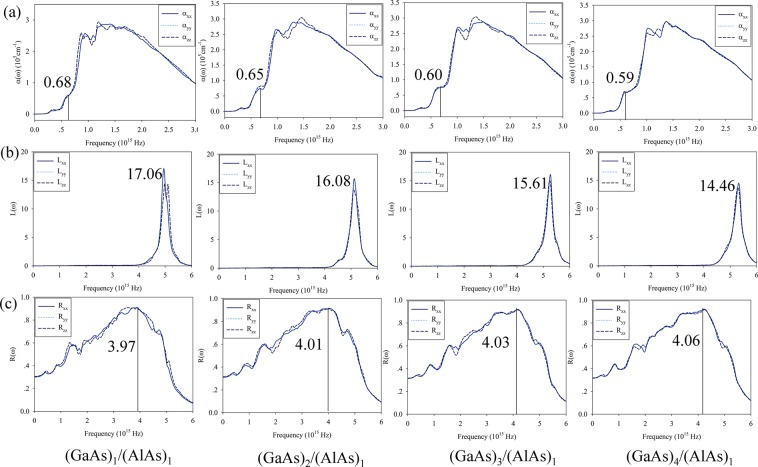
The (**a**) absorption spectra α (ω), (**b**) electron energy loss spectra L (ω) and (**c**) reflectivity spectra R (ω) for (GaAs)_m_/(AlAs)_1_ (m = 1 to 4) SLs as a function of electromagnetic wave frequency.

Figure 4 illustrates the optical properties of GaAs/AlAs SLs as a function of electromagnetic wave frequency. The absorption coefficient of GaAs/AlAs SLs is shown to be anisotropic, due to the reduction in symmetry (see Fig. 4(a)). The first peak of absorption spectra for (GaAs)_1_/(AlAs)_1_ SL appears at $0.68\times {10}^{15}$ Hz. As the layer number of GaAs increases from 1 to 4, the first peak decreases gradually to $0.59\times {10}^{15}$ Hz. Obviously, increasing the GaAs layer number causes a red shift of the absorption spectra for GaAs/AlAs SLs, which will benefit for their application as near infrared detector device. The properties about the elastically scattered and non-scattered electrons could be determined by the electron energy loss spectrum (EELS), and the results are illustrated in Fig. 4(b). The prominent peak of bulk GaAs is around $4.11\times {10}^{15}$ Hz, which is comparable with the value of $4.04\times {10}^{15}$ Hz reported by Ma *et al*.^31^. The peak of EELS for AlAs is located at ~$4.03\times {10}^{15}$ Hz. As shown in Fig. 4(b), the peaks of EELS for GaAs/AlAs SLs shift to lower energies and the peak intensity decreases significantly from 17.06 to 14.46 with the layer number of GaAs increasing. The reflectivity spectra of GaAs/AlAs SLs are further compared in Fig. 4(c). Similar to the absorption spectra and EELS, the reflectivity spectra for GaAs/AlAs SLs are anisotropic, as shown in Fig. 4(c). Clearly, the reflectivity shows a pronounced maximum at $\sim 4.0\times {10}^{15}$ Hz for all considered SLs. However, the layer number of GaAs has negligible effects on the reflectivity spectra of SL structures, and the spectra of all SLs are quite similar to each other. Lou *et al*. employed macroscopic infrared dielectric tensor to study the reflectivity of this SL structure, and found that the SL structures resemble an anisotropic uniaxial crystal^19^, which are consistent with our results. The results indicate that the layer number of GaAs has eligible effects on the reflectivity spectra of GaAs/AlAs SLs, while the absorption coefficient shows a red-shift with the increasing layer of GaAs. The presented study will advance the fundamental understanding of the optoelectronic properties of GaAs/AlAs superlattices for its application as near infrared detector.

## Conclusions

In this study, the electronic and optical properties of GaAs/AlAs superlattices are explored by the density functional theory calculations with reverse scissor correction. The band gaps for (GaAs)_m_/(AlAs)_m_, (GaAs)_1_/(AlAs)_m_ and (GaAs)_m_/(AlAs)_1_ (m = 1 to 5) SLs are all direct. It is also noted that the reverse scissor corrected band gap of (GaAs)_m_/(AlAs)_1_ SLs decreases obviously with the increasing number of GaAs layer, and the corresponding cut-off wavelength all are located in the near infrared region. There is a red-shift for the absorption spectra of (GaAs)_m_/(AlAs)_1_ superlattices with the layer of GaAs increasing, which is beneficial for their applications as near infrared detector. Besides, the maximum values of reflectivity of all SLs are much larger than those for bulk states. This study shows that the stacking periodicity of GaAs has remarkable effects on the electronic and optical properties of GaAs/AlAs SLs and varying the layer number of GaAs/AlAs SLs can be used to tune these properties effectively.

## Methods

All the DFT calculations are implemented in Vienna *Ab Initio* Simulation Package (VASP)^32^. The ion-electron interactions are treated by the projector augmented-wave pseudopotentials^33,34^, and the local-density approximation (LDA) in the form of Ceperly-Alder^35^ is employed to describe the exchange-correlation functional. The convergence criteria for total energies and forces are 10^−5^ eV and 10^−5^ eV/Å, respectively. A cutoff energy of 500 eV and a 4 × 4 × 4 k-points are employed in these calculations. Three types of GaAs/AlAs SLs, i.e., (GaAs)_m_/(AlAs)_m,_ (GaAs)_1_/(AlAs)_m_ and (GaAs)_m_/(AlAs)_1_ (m = 1 to 5), are considered. Figure 1 illustrates the geometries of the representative (GaAs)_2_/(AlAs)_2_ SL.
